# “It feels like medically promoted disordered eating”: The psychosocial impact of gestational diabetes mellitus in the perinatal period

**DOI:** 10.1371/journal.pone.0288395

**Published:** 2023-07-21

**Authors:** Madeleine Benton, Sergio A. Silverio, Khalida Ismail

**Affiliations:** 1 Department of Psychological Medicine, Institute of Psychiatry, Psychology and Neuroscience, King’s College London, London, United Kingdom; 2 Department of Women & Children’s Health, School of Life Course & Population Sciences, Faculty of Life Sciences & Medicine, King’s College London, London, United Kingdom; University of Stirling, UNITED KINGDOM

## Abstract

**Background:**

The global prevalence of gestational diabetes mellitus (GDM) is increasing, and it can significantly impact women’s psychosocial outcomes in the perinatal period. The aim of this study was to explore the psychosocial impacts including experiences of support for women with GDM in the antenatal and postnatal period.

**Methods:**

Semi-structured individual interviews were conducted with women (n = 33) living in the UK, who were either pregnant and recently diagnosed with GDM or had a previous GDM diagnosis within the past three years. Interviews were recorded, transcribed, and analysed using reflexive thematic analysis.

**Findings:**

Analysis revealed six themes: 1. Diagnostic related frustration; 2. Impact on mental health; 3. The medicalisation of eating; 4. Losing agency to gain control; 5. Sourcing networks of support; 6. Current pregnancy; and future reproductive health. Each theme provides a unique insight into the experiences and psychological strain associated with GDM. From confusion and frustration at diagnosis, to the profound knock-on impact a diagnosis, associated lifestyle changes and medical appointments had on women’s mental health, and the perceived medicalisation of their eating behaviours and patterns.

**Conclusion:**

Given the increasing prevalence of GDM and its wide-ranging psychosocial impacts, this study emphasises the need for healthcare professionals to consider the potential implications of GDM on women’s psychosocial outcome, and to consider alternative support options outside of the medical system, such as peer support.

## Introduction

Gestational diabetes mellitus (GDM) is a form of diabetes which occurs for the first-time during pregnancy, affecting around 15% of women globally [1]. The most frequently reported perinatal consequence of GDM is macrosomia (a neonate weighing over 4 kg) which can increase the risk of caesarean section, shoulder dystocia, instrumental birth, and birth injury [2]. GDM typically resolves after birth however, it can have long term consequences for both mother and baby including increased risk of type 2 diabetes in later life [3].

GDM is typically diagnosed using blood glucose levels from an oral glucose tolerance test. The global prevalence of GDM is increasing, due in part to increasing maternal age, obesity, and testing practices. However, the rise may also be due to new diagnostic criteria recommended by the International Association of the Diabetes in Pregnancy Study Groups (IADPSG) which utilise lower glucose cut-offs to diagnose GDM [4]. These criteria have been adopted by some countries, but not others, leading to variations in GDM diagnosis based on location. Despite a rise in GDM prevalence following the introduction of the new IADPSG criteria [5], pre-post studies have shown minimal clinical improvement in adverse outcomes [6].

Management of GDM is demanding on women and involves self-monitoring of blood glucose, diet and exercise modification, and in some cases the use of pharmacological treatment including metformin and insulin [7]. Women will have increased contact with health care professionals (HCPs) and the intensive management of GDM has the potential to change the contextual experience of pregnancy from ‘normal’ to one that is highly medicalised [8].

A growing body of literature has demonstrated the impact of GDM on women’s mental health outcomes. Qualitative research has highlighted psychological distress, guilt, shame, and self-blame experienced by women with GDM at different stages of the pregnancy [9, 10]. Furthermore, more research suggests the association between GDM and the subsequent development of mental health symptomatology, notably depression and anxiety. A recent meta-analysis reported women with GDM are 2–4 times more likely to develop depression in the antenatal or postnatal periods in comparison to those without GDM [11]. Research has often been conducted in the antenatal period, with limited attention paid to the ongoing psychosocial experiences and consequences of GDM. It is important to understand the impact of GDM through a woman’s whole pregnancy, birth, postpartum period, and psychosocial support throughout, to gain a comprehensive understanding of the impact of GDM.

The aim of this study is to explore the psychosocial impacts including experiences of support for women with GDM in the antenatal and postnatal period.

## Methods

### Study design

This qualitative analysis forms part of a wider study with a primary aim of understanding the impact of GDM on the mother-infant relationship. This analysis pertains to women’s psychosocial experiences of GDM in the antenatal and postnatal period. All women expressed considerable insights into their experiences of care, support, and impact on mental health which had important consequences for experiences of pregnancy and postpartum health, which warranted closer examination. This allowed for a secondary analysis, in addition to that which was first intended and is to be reported elsewhere. COREQ (COnsolidated criteria for REporting Qualitative research) reporting guidelines were followed and COREQ Checklist completed.

### Ethical approval

Ethical approvals were sought and granted from King’s College London Research Ethics Committee (reference number: HR/DP-21/22-26417). All participants provided written consent prior to all interviews.

### Study team and theoretical perspective

The research team is multi-disciplinary, comprising a research associate (MB), psychologist (SAS), and a psychiatrist (KI), with expertise in women’s mental health (MB, SAS), and diabetes (MB, KI). The philosophical underpinning was seated in critical realist ontological and objectivist epistemological domains [12] and positionality comprised a critical (empathic) approach to reflexivity and an objective outsider position within the data (as none of the study team had experienced GDM). Together, these made for a post-positivist research paradigm [12], whereby participants’ narratives were accepted as ‘truths’ or ‘lived realities’.

### Recruitment, setting, and participants

This study was conducted in the United Kingdom. Recruitment took place on-line via advertisements distributed on social media to relevant groups (including support groups for GDM, pregnancy support groups, and pregnancy and postpartum support group from ethnically diverse groups). Inclusion criteria were: i) being pregnant with a diagnosis of GDM or having a diagnosis of GDM in the past three years, ii) at least 18 years of age, and iii) able to read and speak in fluent English. Interested participants e-mailed the research team and received a participant information sheet and consent form to be completed before or at the beginning of their interview. Purposeful sampling was used to recruit a diverse group of women, including those at different stages of pregnancy, with varying numbers of children, and from different ethnicities [13]. Interviews were scheduled at a time that was convenient for women.

Women (n = 33) were interviewed and ranged in age between 28–42 years (*M*_*Age*_ *=* 34 years). Most women were in the postnatal period (n = 24), with the remainder being pregnant (n = 9), with fewer reporting being multiparous (n = 9). In relation to ethnicity, women self-identified as White British (n = 20), White other (n = 5), Mixed Asian (n = 2), Caribbean (n = 1), Indian (n = 1), Japanese (n = 1), Pakistani (n = 1), and Sri Lankan (n = 1). Women were living in different areas of the United Kingdom.

### Data collection

The first author conducted individual semi-structured interviews, which provided a standardised structure, while allowing for flexibility in exploring topics of interest in more depth [14]. The interview schedule was piloted with one woman who had GDM prior to the interview, this interview was not included in the analysis. Questions were augmented as the number of interviews progressed, depending on themes which were being identified and content of interviews. Example questions include: How did you feel when you were told you had GDM? How do you think your diagnosis of GDM impacted how you felt about your pregnancy?

Interviews were conducted virtually between January and March 2022 using video-conferencing software and ranged between 25–61 minutes (*M*_*Time*_ *=* 39 minutes). With consent, interviews were recorded.

### Data analysis

Audio recordings of the interviews were transcribed verbatim, anonymised, and imported into NVivo v.12 for data management and analysis. Data were analysed using inductive reflexive thematic analysis, which offers theoretical independence/flexibility for a wide variety of qualitative data. The six phase process of reflexive thematic analysis [15] was followed, which in brief entails familiarisation, generating initial codes, searching for and then reviewing themes, defining and offering names for those themes. Themes were developed by the first author and discussed and refined with all authors. Sample size sufficiency, data adequacy, and theme saturation were assessed during the analysis process using existing models of thematic concordance and data quality [16, 17]. This meant data were fully saturated with the number of participants recruited, and common themes could be found across the dataset, with no new themes being generated by the time the last transcript was analysed.

## Results

**Fig 1:**
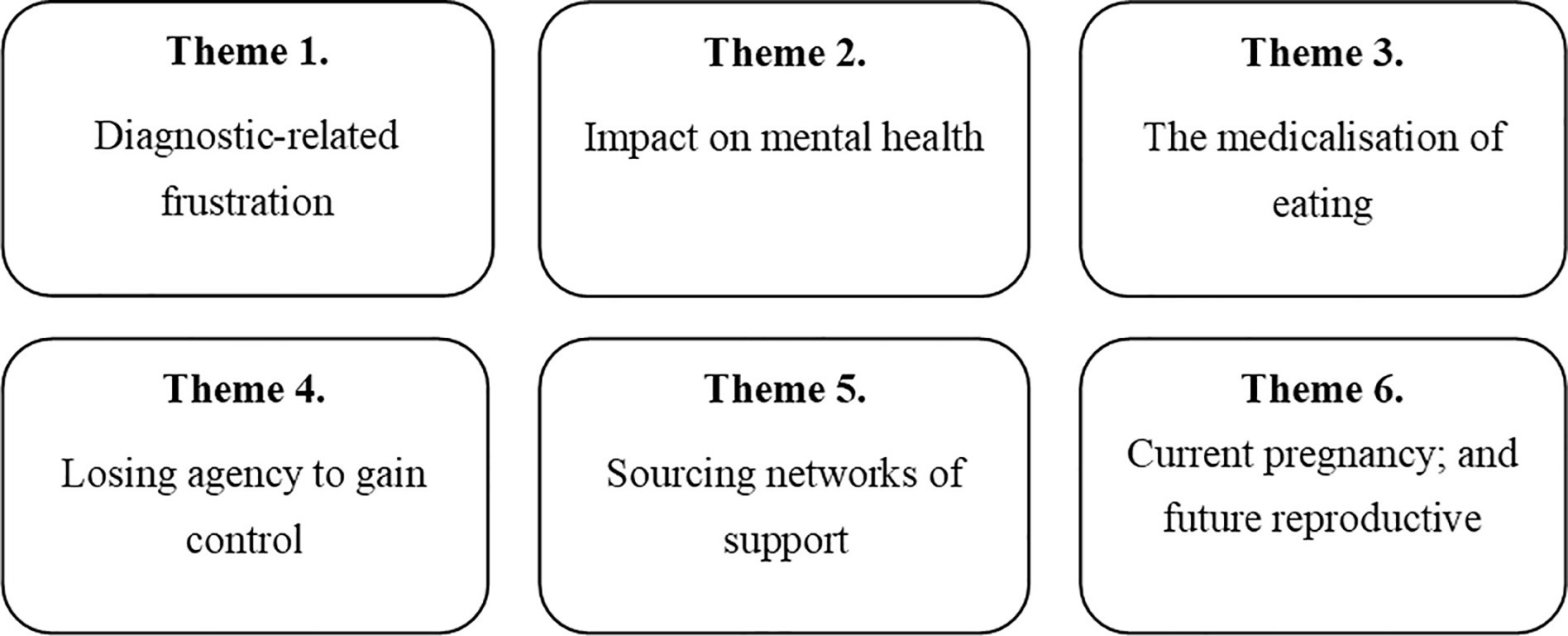
Psychosocial impact of GDM in the perinatal period.

Analysis resulted in six overall themes related to the psychosocial impact of GDM in the perinatal period (Fig 1). Quotations from the participants are presented using best exemplars to illustrate the themes.

### Theme 1

#### Diagnostic-related frustration

This theme describes women’s initial reactions, thoughts, and feelings surrounding diagnosis of GDM. Almost all women described initial shock and disbelief after learning they had GDM. The diagnosis appeared to be more difficult to accept for women who did not consider themselves ‘at risk’ of developing GDM.

“I was pretty devastated I’m not going to lie… I kind of probably falsely assumed that it was more for people who were severely overweight or extremely unhealthy throughout their pregnancy and I didn’t feel like I had been… I was pretty shocked.” (P19:postnatal)

Frustration was often connected to issues surrounding communication of the diagnosis, diagnostic thresholds, and dietary advice received from HCPs. Women reported a range of experiences with regards to the communication of the diagnosis and management information, including feelings of being overwhelmed by the amount of information received, delays in the communication of results after testing, or by the number of HCPs involved.

“That first appointment I had, I come out more confused than anything else.” (P11:postnatal)

“I was expecting somebody to kind of talk to me there, but really all they did was give me a blood glucose monitor and say do this four times a day and then they gave me a link with a video and some generic information.” (P8:antenatal)

For women who had GDM in a previous pregnancy, receiving a diagnosis of GDM again was often anticipated and thus, was somewhat easier for them to accept compared to their initial diagnosis.

“I think in the later pregnancies, I’d expected it… the first one when I was told, it was very much being hit for a six… much more a situation of confusion, not understanding what was going on.” (P20:postnatal)

Diagnostic thresholds for GDM were a source of frustration for many women. Some women felt they were being diagnosed with GDM just because of the lower diagnostic thresholds within the hospital or healthcare trust providing their care. A few women were informed their results were ‘borderline’, which contributed to feelings of uncertainty regarding the necessity of treatment and confusion about how to manage the condition.

“Through the research I’ve done, I realised that maybe if I lived in Ireland, I wouldn’t have gestational diabetes. If I had a different Trust, maybe I wouldn’t have that diagnosis.” (P1:antenatal)

Alongside feelings of guilt and shame, women described a sense of failure, feeling let down by their bodies and blaming themselves for the diagnosis. Women also described disappointment, fear for their baby’ health, and feeling like they had lost control.

“It made me feel like my body wasn’t capable of growing and supporting a baby effectively, that there was something wrong with my biology and it was just all breaking down.” (P1: antenatal).

“The biggest thing when I received the diagnosis was that, oh my gosh, I’ve done something that’s going to harm my baby, it’s my fault, quite a lot of self-blame and it is still there in the back of my mind, like that mum shame.” (P8:antenatal)

### Theme 2

#### Impact on mental health

Women described considerable strain of GDM on their mental health. Many described management as relentless, exhausting, burdensome, and a stress-inducing process. Some even compared the management of GDM to a full-time job and reported that they were unable to relax and enjoy their pregnancy.

“I was thoroughly miserable… I was horrible to be around, very irritable, I felt really low. I felt like I couldn’t relax ever, because I had to time when I ate, and I had to time when I did my finger pricks and had to think about all the medications … I was really stressed and really, really low.” (P4:postnatal)

“Looking back, I was so exhausted by the time I had her mentally. I had nothing left and then I had a new baby.” (P12:postnatal)

Women expressed disappointment in the lack of awareness and acknowledgement of the impact of GDM on their mental health, primarily from HCP’s.

“…the focus I felt, was very much just on the numbers and without any real kind of consideration of what all these changes and what the kind of constant threat of type 2 diabetes or going on more medication feels like for the person going through it.” (P19:postnatal)

One woman remarked that being reassured her baby was fine made her feel selfish for disclosing that she was struggling with her mental health.

“When I mentioned to them [HCPs] I was struggling and finding it difficult mentally. It was always…‘but you’re doing so well’, and ‘baby is doing really well though’… I don’t think there’s really much appreciation of the kind of toll it takes on you… it makes you feel a bit selfish for saying ‘OK baby it’s fine but I’m not so fine’.” (P9:postnatal)

The experience of GDM was made more difficult for women who have experienced previous pregnancy complications or mental health problems, but for these women mental health history was rarely discussed.

“I’ve been anxious most of my life… but with this pregnancy it was just like a whole new level… when I had the gestational diabetes test, it all came flooding back… I was having such bad intrusive thoughts, just all about the baby not making it…I wouldn’t go out because then I felt like I’ve missed feeling the baby move… the GDM just made it spiral, ’cause I was so worried.” (P12:postnatal)

“… you hear of gestational diabetes associated with like poorly babies, early babies, sometimes still birth, and I’ve had pregnancy losses before, so I think I went straight to that dark place of something terrible is going to happen.” (P4:postnatal)

Some women reported attempting to communicate their mental health concerns yet felt ignored or required considerable self-advocacy to receive support. One woman felt HCPs were deterring her from accessing mental health services:

“…if I brought it up, any kind of problems with my mental health, it was kind of, ‘so we can put that into the system, but if I do that it’s gonna open-up this whole box of different questions that we have to go through. Do you want to do that?’ And kind of the undertone is like maybe don’t do that… I definitely got the impression that they would rather…I didn’t push for anymore kind of support in that sense.” (P19:postnatal)

Women emphasised the importance of considering other maternal and child health outcomes beyond labour and birth, including mental health.

“To make sure that you have as many mothers and babies survive birth, that’s a minimum standard. But don’t forget that there are other… outcomes that we’d like to see, like positive birth experiences, positive bonding with mother and baby, breastfeeding initiation rates being higher…those things are also important as well as just, ‘you’ve got your baby that’s all that matters’. You know, some women are left like traumatised.” (P1:antenatal)

### Theme 3

#### The medicalisation of eating

Dietary changes required for managing GDM had psychological effects on women, including anxiety and stress about food choices and how food would affect blood glucose, and health of their baby. Changes to their diet also disrupted some women’s relationship with food and triggered previous eating disorders. Food consumption was discussed as a centre point of management and women’s daily living.

“To think about every single thing you put in your mouth and for that potentially to harm your baby, it’s just an absolute minefield.” (P26:postnatal)

“There was a lot of… eating disorder associated thoughts and feelings, where there is a lot of fear before testing blood sugars… I was really pre-occupied for a long time about what will I eat? When will I eat? How is that going to affect my blood sugars?… will I have to go on medication? How is that going to affect my birth choices etc?” (P14:antenatal)

“It just feels like it’s kind of like medically promoted disordered eating” (P19:postnatal).

Women expressed considerable frustration with the dietary advice received, and felt it was unhelpful and inadequate. Women highlighted that they were often outdated, and provided recommendations that were unhelpful in managing blood glucose.

“The diet recommendations, I cannot fathom. I can’t fathom how or why they are the way that they are…” (P1:antenatal)

Women express frustration with the lack of understanding of GDM among family and friends. In particular, the extent and burden of diet changes as well as the associated risks for them and their babies.

“I think being able to eat with family and getting people to understand. I think that was my big thing, when people would say, “Oh just have a little bit, or just one meal won’t hurt” but I was like actually, I don’t know that. One meal might hurt… might be all I need to flow my levels up to then get me on either Metformin or Insulin… but no one seems to understand… one meal probably wasn’t gonna, suddenly cause him to be huge or cause a still birth but the risk was still there the whole time, I was still carrying the worry of that with me.” (P13:postnatal)

### Theme 4

#### Losing agency to gain control

Women described considerable anxiety and stress related to managing blood glucose levels. A ‘3 strike’ system was discussed where if they were not ‘achieving’ optimal blood glucose levels they had ‘failed’ and there would be a need for insulin therapies.

“I’m getting these red readings that are going through to the hospital… they said three strikes… you’re out… you’re onto medication… my anxiety was through the roof, and I felt sick…what score is this going to give me? And that’s going to go straight through to the diabetes team … I thought they’re going to come back and put me in a strait-jacket… strapping me up and taking me away… I just felt like I was defeated.” (P1:antenatal)

Women perceived the transition of management from diet ‘controlled’ to insulin therapy as a failure. Women were fearful of the impact of insulin on their baby and birth choices. This fear was often in-turn a driver for strict management through diet.

“…the bit… I found hardest is that sort of like, I need to manage this, otherwise I’m going to be defeated and have to go on medication and that for me was…a psychological kind of marker of like failure.” (P21: antenatal).

Some described appointments and conversations with HCPs as a ‘constant battle’, especially when discussing birth options. Women described ‘psyching themselves up for appointments’ which was draining, stressful, and took the enjoyment out of pregnancy.

“It was just that kind of constant battle that I thought, as professionals, you should be there to help and advocate for me… it was… quite tense going to appointments, there wasn’t that kind of joy. It was a bit like, well, what’s the argument going to kind of be today?… rather than kind of going to those appointments and being like, oh, I’m going to see her [baby]… there was this cloud that was over it all.” (P11:postnatal)

One woman suggested, fear of HCP’s can lead to women withdrawing from the medical system:

“For people like me, [HCPs] cut me off from the system, so when something might go wrong, I’m less likely to put my hand up, because I am already thinking, you’re gonna lock me up if I get a red reading…I’m already thinking, I’ll just take the risk thanks, because I don’t feel like I can talk to you as an adult helping to make informed choices together. I feel that you will, through a lot of risk language, you will really coerce me, so I’ll…stay quiet.” (P1:antenatal)

Women expressed disappointment in the lack of birth options, and lack of communication and decision making around them.

“So, I’ve been told that I’ll have to give birth on a labour ward versus the midwife led unit, although I’m sure I do have, I know I do have some choice in that, but this is how it’s communicated to me. ‘You need to give birth in the labour led unit’.” (P8:antenatal)

“It was a bit like now you’ve got gestational diabetes, so actually you need to be induced. But there was no conversation about actually with induction comes X, Y and Z.” (P11:postnatal)

### Theme 5

#### Sourcing networks of support

Women described mixed feelings around the transition from their community midwife to the diabetes team. Some felt it was disjointed, stressful, and felt abandoned by their community midwife. Women described considerable numbers of HCPs that were involved in their care which was often overwhelming.

“Every time I went to an appointment, I saw someone different. So, there was not necessarily that continuity of care, I had that to a certain extent with the community midwife, but again, I think as soon as she found out about gestational diabetes she was like, so I probably won’t see you again then, because you’ll be under that team.” (P11:postnatal)

“I saw a different person every single time… a different consultant every single time, a different diabetes person, so like people didn’t know me… (P12:postnatal).

One woman who was able to see the same midwife at each appointment, highlighted how this was beneficial:

“I think I was one of the only people that managed to see the same midwife at every antenatal appointment… it made the experience so much better.” (P25, postnatal)

For some women a lack of social support led to feelings of isolation.

“I had no support or help from anyone and even from friends and family as well because no one had GDM, that I knew of. So, you’re left in a in a dark place where no one around you can help you”. (P29:postnatal)

Women described the importance of a supportive partner in reducing the psychosocial burden of GDM. Women’s partners’ also modifying their diet alongside the women offered emotional and practical support.

“…luckily, I also have a very supportive husband who’s been very proactive in helping me to maintain the diet…he would go out and do all the shops to make sure that we have the foods that are good for me…without having that additional level of support, it would have been much harder to maintain a diet-controlled GDM.” (P14:antenatal)

In response to frustration with NHS dietary advice, women sought support from online peer groups. Peer support groups were described as central to improving women’s experience of GDM, as well as a primary source of information surrounding GDM, especially dietary advice.

“The [Facebook] page was massively helpful and actually was where I got most of my advice” (P18:postnatal).

Hearing other women’s experiences with GDM was highly valued and helped reduce feelings of self-blame, guilt, shame, and isolation.

“I found the Gestational Diabetes UK group on Facebook… that’s the thing that really helped the most because no one else understands in your life… So just having that support group was so helpful… It’s just that people understanding what you’re going through in that moment.” (P24:postnatal)

“…had I not found that gestational diabetes group on Facebook, where would I have asked all my questions? Like where would I’ve got any reassurance from people or knowing that birth stories can still be positive.” (P13:postnatal)

### Theme 6

#### Current pregnancy and future reproductive health

The postpartum impact of GDM was varied. Some women reported feeling relieved to no longer have GDM after giving birth. Many celebrated their ability to eat foods they hadn’t been able to during pregnancy, however, this was often short-lived.

“…we came home from the hospital and were like, right. I’ve had my chocolate. I need to be sensible ’cause I’ve got the increased risk of diabetes type 2 and we need to set good examples for the children.” (P4:postnatal)

The significant lifestyle changes made during pregnancy often made it hard for others to ’switch off’.

“You’re just constantly on high alert… How do you just switch that off? You can’t just switch it off.” (P13:postnatal)

Despite being less concerned about GDM than in pregnancy, many women continued to worry about the increased risk of type 2 diabetes in later life for themselves and their baby.

“It does sit in the back of your mind there’s probably times when he [baby] has sort of been close to me and breathed and I thought oh my god…does his breath smells sugary?…is he weeing more than usual? … you start thinking through symptoms of diabetes.” (P13:postnatal)

In the weeks leading up to their annual tests, many women expressed anxiety that they may have developed type 2 diabetes.

“I’d like to say that I’ve just forgotten about… moved on. But, I haven’t. I’ve still got the lurking in my mind of when I have to do the next test… ’cause obviously I’d rather not have developed type 2 diabetes.” (P19:postnatal)

For some women, feelings of guilt surrounding the impact of GDM on their infants continued in the postpartum period.

“… there are definitely some feelings of guilt, and I’ve brought him [baby] into the world with this already heightened risk, which no one wants a diabetes diagnosis, so I feel bad about that.” (P19:postnatal)

Postpartum, GDM had a lasting impact on some women’s relationships with food, which in turn affected their thoughts about feeding their babies. Some multiparous women reported that this was not an issue with their other children from non-GDM pregnancies.

“It has left the mental health impact of just being constantly over aware of what I’m eating… I spent the last three months constantly checking how many carbohydrates everything has. And turning that switch off is really difficult… I still have that voice in the back of my head saying maybe this is too high carbohydrate, this might increase your risk of diabetes. Should you have this?” (P19:postnatal)

“I wouldn’t say I have an eating disorder from it, but I would certainly say it’s caused some disordered eating habits… I completely put it down to the fact that you are so obsessive, and perhaps that was my failings in that I didn’t want to go on some medication. So I was very…strict. I could have maybe been less strict with it, but then risked the medication” (P13:postnatal)

GDM had an impact on some women’s future family planning, with some deciding not to have more children due to the negative effects of GDM on their pregnancy experience and increased likelihood of developing GDM in future pregnancies.

“In an ideal world I would have more children, but after having gestational diabetes, I just wouldn’t.” (P26:postnatal)

Many women were aware that they would likely be tested earlier for GDM in future pregnancies and would therefore have a longer time to manage their condition.

“I did it [manage GDM] for… 12 weeks and… that was really hard. But with your next one… it starts earlier…so then I’m thinking gosh like if I’m getting it from 16 weeks, from 12 weeks, from 8 weeks [gestation]… long time to manage a diet which has impacted my relationship with food but also then all the medication side of things, which we had already tried so hard to avoid, there’s no guarantee that I can do that again.” (P13:postnatal)

Some women were aware of the increased risk of developing GDM in subsequent pregnancies, but they felt that their previous experience had prepared them better. Some planned to change their diet before conception and monitor blood glucose earlier in pregnancy before a formal diagnosis was made.

“Although it was a negative experience at the time, I think it’s taught me a lot. I’m planning to get pregnant again, hopefully, and I recognise that I’ve got around a 50% chance of developing it in a second pregnancy….I feel like I’m armed with knowledge now so I would cope with it very differently.” (P33:postnatal)

Women reported mixed experienced with continuing lifestyle change postnatally. Some formed ’new habits’ during pregnancy, and continued these changes, such as diet modification, while other cited children, and sleep deprivation as barriers to continued lifestyle change. Women reported motivation, namely their baby, for making drastic changes to their diet during pregnancy, often faded after giving birth.

“When you do it, you do it with your baby in mind. It’s fine, but actually it was harder to stick to…when I wasn’t pregnant with him, because I didn’t need to do it.” (P13:postnatal)

Women described yearly tests for type 2 diabetes, as a motivating factor to eat more consciously.

“…because I have my yearly HbA1c it’s like…no other diet ’cause there is like an outside force checking up on you… it’s motivational.” (P33:postnatal)

Women discussed little follow up after baby was born. Women felt like had the knowledge they were at increased risk to developing type 2 diabetes, without any real support to continue lifestyle changes and no reminders to do their yearly HbA1c test.

“…you’re just left with, “you’re at higher risk, who knows, good luck” Like there’s no, there’s no support.” (P13:postnatal)

## Discussion

Analyses of these data showed six distinct themes could be derived from the experiences of GDM by women in the UK. These themes coalesced around a central organising concept: ‘The psychosocial impact of GDM in the perinatal period’. Each theme, in-turn, provided a unique insight into the experiences and psychological strain associated with GDM, from the very start when first diagnosed causing confusion and frustration, to the profound knock-on impact a diagnosis, associated lifestyle changes and medical appointments had on women’s mental health, and the perceived medicalisation of their eating behaviours and patterns. Furthermore, many women reported having to counter the negative by sourcing their own networks of support in the absence of friends’ and familial understanding, whilst analysis also elucidated women’s fears about their future life course and reproductive health given their GDM diagnosis in their current or last pregnancy. The central organising concept therefore is not only derived from the themes, but neatly draws them together for discussion within the wider literature-base, which follows below.

Descriptions of initial shock, disbelief, self-blame, and guilt after learning they had GDM are in line with previous research [10]. In our study a GDM diagnosis appeared to be more difficult to accept for women who did not consider themselves ‘at risk’ of developing GDM. Previous research has reported that women who were able to explain the cause of GDM were able to process and accept the diagnosis more readily than those who had little understanding of GDM [10]. Our findings around frustration with diagnostic thresholds, are interestingly concurrent with previous research conducted in the UK over 10 years ago, which reported hospitals did not have uniform diagnostic thresholds, and women felt after being told their condition was ‘borderline’ that treatment was unnecessary [18]. Recent research has suggested the exploration of a model of care based on stratification or individual level of risk for pregnancy and birth complications for women with GDM [10] with the aim to reduce the need for all women to be labelled as having GDM and negating unnecessary anxiety and distress which has been consistently reported by women.

Women discussed considerable stress and anxiety associated with GDM and disappointment in the lack of awareness of the impact of GDM on mental health. These findings are concurrent with a recent meta-analysis which reported women with GDM were 2–4 times more likely to develop depression in the antenatal or postnatal periods in comparison to those without GDM [11]. However, previous research has focused on the examination of depression and anxiety with little assessment of other mental health outcomes for example eating disorders and stress-related disorders. It is also important to note the considerable body of research highlighting the impact of maternal stress and anxiety on infant development. Specifically, studies have highlighted the increased risk of neurocognitive disorders, diabetes, and cardiovascular disease in babies whose mums experienced considerable stress during pregnancy [19–21]. Our findings highlight from women’s perspectives a gap in current care in terms of the impact of GDM on mental health. Women expressed a need for increased awareness, and additional support, particularly for women with experienced mental health difficulties prior to pregnancy.

Anxiety and stress surrounding food choices during pregnancy impacted on some women’s relationship with food and in several cases leading to disordered eating behaviours. Little research has examined the impact of GDM on feeding and eating disorders however interestingly, analysis of the Danish National Patient Register including 19,980 women with GDM found that maternal diabetes was associated with an increased risk of feeding and eating disorders in offspring [22]. A handful of qualitative research has touched on the impact of GDM on eating and feeding behaviours include starvation and purging [8, 18].

Notably, women rarely discussed physical activity in pregnancy and focused on considerable dietary modification. The transition of management from diet ‘controlled’ to insulin therapy was described as a failure. Previous research has highlighted the need for insulin increased the perception of severity of the condition for women, and women described the progression to insulin as a constant threat, resulting in anxiety and fear returning, comparable to when first diagnosed [18, 23].

In line with our findings, previous research has reported disappointment and isolation expressed by women when they perceived a lack of healthcare system support [10]. A key finding of our research was the importance of online peer support groups, which reduced feelings of isolation for women and was an important source of support external to HCPs. Peer support appears to be incredibly acceptable to women, improving their overall experience and could be an underutilised resource by HCPs. It should be noted that not all women will be able to access online resources and the potential digital divide, may exclude women from accessing such support.

We highlighted the lasting impact of GDM postpartum, which has received less attention in the literature. Notably, most women were aware of the long-term impacts of GDM including increased risk of type 2 diabetes for them and their baby. This is contrary to a previous review reporting low awareness of the long-term risks associated with of GDM [10]. This may be reflective of improved information provision by HCPs around GDM in the UK. Interestingly, negative experiences of GDM impacted on decisions surrounding future family planning which is in line with previous research highlighting negative pregnancy experiences impact on future reproduction [24, 25]. Importantly, for prevention interventions for type 2 diabetes our findings are in line with previous researcher which highlighted continued lifestyle modification to prevent future diabetes appears to dissipate after birth, possibly because the driver to protect their unborn child is no longer there [26].

### Strengths and limitations

Several of our findings are in line with previous research, however it is noteworthy that we highlighted specific impacts on mental health, social support, and the long-term implications of the condition. While a limited number of studies on GDM in UK women have been conducted, these have been conducted in either one geographical/hospital setting [8, 27], or with data collection occurring over 10 years ago [18]. Our use of online data collection enabled us to interview a diverse group of women from various regions of the UK. A key limitation is that the sample was self-selecting and therefore, women who may have been very distressed during pregnancy or had negative experiences may have been more motivated to participate than less distressed women. Additionally, women were recruited through online channels and women recruited from elsewhere may not value this type of social support and may fundamentally have different experiences which mean that they did not feel the need to seek out support. Further, a key limitation which would be legitimate to raise would be the fact that none of the research team were currently seeking care and treatment for GDM, meaning our position within the data was very much as an outsider. This could have introduced some difficulty in understanding participants’ experiences and future research should consider the role of patient and public involvement and engagement [PPIE] to assist in meaning making.

## Conclusion

This study highlights the multidimensional and wide-ranging implications of GDM on women’s psychosocial experiences and outcomes in the antenatal and postnatal period. Although GDM typically resolves after birth, it has long lasting impacts on women. Considering the rising prevalence of GDM and the wide-ranging psychosocial impacts, the findings of this study highlight the need for HCPs to consider the implications that a GDM diagnosis may have on women but also alternative support options outside of the medical system including peer support. It is essential that women diagnosed with GDM receive consistent individually tailored evidence-based information and ongoing psychological and social support. This qualitative analysis has produced evidence demonstrating the clinical care received can have a detrimental impact on women’s pregnancy and postnatal experience and the importance of peer facilitated support for women.
